# Supplementary material for: Cognitive Effects of Reducing First-Generation Antipsychotic Dose Compared to Switching to Ziprasidone in Long-Stay Patients with Schizophrenia
Source: J Clin Med. 2024 Apr 4;13(7):2112. doi: 10.3390/jcm13072112 (PMC11012535; doi:10.3390/jcm13072112)
Supplement: Supplementary file 1 [file jcm-13-02112-s001.zip › jcm-2894147-supplementary.pdf]

Supplementary Table S1. A brief description of the tests used to assess cognitive function

All tests are described in M. Lezak, Neuropsychological Assessment, 3rd ed.; 1995, Oxford University Press, N.Y. Parallel versions of each test are available.<br>Together the tests take about 45-50 minutes to administer. The Trailmaking Test, The Digit Span test, Symbol Digit Modalities Test, Stroop color-word test and Verbal Fluency test each take about 5 minutes and are especially suited for the population in question. The Rey Auditory-Verbal Learning Test and the Continuous Performance Task are more complex, but provide robust data.
**Trailmaking Test, part A and B**
Trailmaking is a test which operationalizes the speed of information processing and set shifting between concepts. Time, measured in seconds, in part A and part B, is the main variable.
**The Digit Span Test (WAIS), forwards and backwards**
The procedure measures short-term memory and working memory. The scores on the forward series and the backward series are the main variables.
**Symbol Digit Modalities Test**
The test measures the speed of information processing in a simple cognitive task. The maximum score in a standard, time-limited, session is the main variable.
**Rey Auditory-Verbal Learning Test.**
A list to be learned consists of 15 meaningful monosyllabic words, which are presented in 5 trials. Each trial is followed with a free recall of the words. After a period of 20 minutes after the end of the 5th trail, each participant is asked to mention as many words as possible ("delayed recall") , followed by a trial of delayed recognition. The test measures the process of retrieval and consolidation of information. The main variables are: A: The sum score of the 5 trials. B. The total number of correct words in the delayed recall trail. C. The total number of correct words in the delayed recognition trial.
**Stroop Color-Word Test**
The test consists of three subtests that measure A. the basic speed of the process of reading. B. The speed of naming of colors. C. The speed of inhibiting the automatism of reading. The test measures selective attention. The main variable is the interference score, whereby the time of subtest 2 is subtracted from the time of subtest 3.
**Verbal Fluency Test**
The test is composed of 4 components: 2 tasks with a time limit in which as many words as possible should be generated (letter N and letter A) and 2 tasks whereby a category must be generated (professions and animals). The score is a measure of the generating of words from the semantic memory. Two main variables: the total score of the letters and the score of the categories.
**Continuous Performance Task**
A computer version (MINDS) to measure the maintenance of a task. The subject must react to a visual target. The main variable is the median of the reaction times.
